# Equipping youth for meaningful policy engagement: an environmental scan

**DOI:** 10.1093/heapro/daz071

**Published:** 2019-07-20

**Authors:** Emily Jenkins, Liza McGuinness, Rebecca Haines-Saah, Caitlyn Andres, Marie-Josephine Ziemann, Jonny Morris, Charlotte Waddell

**Affiliations:** 1 School of Nursing; 2 University of British Columbia, Vancouver, BC V6T 2B5, Canada; 3 Department of Community Health Sciences, University of Calgary, Calgary, Canada; 4 Canadian Mental Health Association, BC Division, Vancouver, Canada; 5 Faculty of Health Sciences, Simon Fraser University, Burnaby, Canada

**Keywords:** youth, engagement, education, mental health promotion, policy

## Abstract

To better address the mental health and substance use crises facing youth globally, a comprehensive approach, inclusive of mental health promotion is needed. A key component of mental health promotion is policy intervention to address the social and structural determinants of health. Importantly, youth should be engaged in these efforts to maximize relevancy and impact. Yet, while there is growing interest in the inclusion of youth in the policymaking process, there is a paucity of guidance on how to do this well. This environmental scan reports findings from a comprehensive search of academic and grey literature that was conducted using the electronic databases: CINAHL, ERIC, MEDLINE, PsycINFO, Google Scholar, and Google. Search terms included variations of ‘youth*’, ‘educat*’, ‘engage*’, ‘policy’ and ‘policy training’. Thirteen English language training programmes met inclusion criteria. Analysis identified marked differences in programme philosophy and focus by geographic region and highlights the need for enhanced evaluation and impact measurement moving forward. This paper makes a needed contribution to the evidence-base guiding this key mental health promotion strategy, which holds the potential to address critical gaps in approaches to youth mental health and substance use.

## INTRODUCTION

Developing effective strategies to tackle the mental health and substance use crises facing ‘youth under 24 years of age’ is global priority (World Health Organization, 2014). An estimated 10–20% of the youth population experience mental health disorders and an even greater segment are living with sub-clinical or undetected mental health challenges (World Health Organization, 2018). Similarly, problematic substance use among youth (i.e. early onset, frequent use, substance use disorder) is a leading public health concern, with youth four times more likely than adults to report related harms (Rotterman and Langlois, 2015). Adding to the burden, <20% of youth experiencing mental health and substance use challenges receive the services they need. This is a stark treatment shortfall and a violation of youths’ human rights (Waddell *et al.*, 2014). Given that the majority of mental health and substance use challenges first arise in childhood and adolescence—with high likelihood of chronicity—youth-targeted interventions are critical to improving health, social and economic outcomes throughout the life course. 

To address this health challenge, researchers and mental health advocates have long argued for a population health approach—incorporating promotion, prevention and treatment within a ‘healthy public policy’ framework (Waddell *et al.*, 2014). Healthy public policy is characterized by ‘explicit concern for health and equity in all areas of policy and by an accountability for health impact’ (World Health Organization, 2018). Yet while much research has focused on the prevention and treatment of youth mental health challenges, there has been a limited focus on mental health promotion to date. Mental health promotion focuses on enhancing positive mental health for all people—including groups identified as experiencing mental health challenges or risk as well the general population (Clarke *et al.*, 2015). Mental health promotion involves strategies to strengthen individuals and communities, and to reduce structural barriers (e.g. socioeconomic disadvantage, discrimination) so that populations have the capacity and resources to optimize their mental health (Barry and Jenkins, 2007).

Although there is an extensive literature on ‘mental health interventions’—both prevention and treatment—‘mental health promotion’ evidence specific to youth is still only emerging and has not yet focused at a policy level (Barlow *et al.*, 2003; Browne *et al.*, 2004). For example, systematic review findings of youth mental health promotion interventions describe effective programmes as characterized by the use of theory and needs assessment data, participatory design and multi-level interventions with built in strategies for implementation and sustainability (Barlow *et al.*, 2003; Barry and Jenkins, 2007; Clarke *et al.*, 2015). Furthermore, while there is considerable literature on the policy and practice reforms needed for enhancing youth mental health and substance use outcomes, this literature is primarily focused on system-level transformation, with an emphasis on integration and collaboration across prevention and treatment service providers and sectors, therefore not addressing mental health promotion (e.g. Hoagwood *et al.*, 2001).

Healthy public policies were defined as ‘key health promotion actions’ within the World Health Organization’s ‘Ottawa Charter’ (Kemm, 2001). Encompassing health and social services, healthy public policy prioritizes the ‘upstream’ domains that comprise the social and structural determinants of mental health and substance use for youth, such as adequate housing, opportunities for healthy child development, sustainable livelihoods, safe neighbourhoods and other community resources (Milio, 2001). These social and structural determinants have also been deemed as fundamental human rights for youth, as defined by the United Nations’ ‘Convention on the Rights of the Child’. Adopted in 1990, the ‘Convention’ is the most ratified international human rights treaty in history, and emphasizes youths’ rights to ‘provision, protection and participation’ including freedom from poverty, discrimination, violence and other harmful social conditions that limit their ability to learn, thrive and grow to their full potential [(Pledger-Fonte, 2012), p.1457)]. The right to protection from harmful health and social inequities and the right to participate and have a voice in the policies that affect their lives is also at the core of suggested healthy public policy frameworks (Milio, 1986, 1988).

Beyond the research context, global interest in ‘youth civic engagement’—or involvement in activities that affect young peoples’ lives, including participation in policy processes, is growing. The UN World Programme of Action for Youth (WPAY) affirmed the full and effective social and political engagement of youth, including engagement in economic, social and political decision making, as one of its 10 priority calls for action (United Nations, 2010). The call explicitly recognizes youths’ ability to contribute solutions to the social issues they face and supports efforts to build youth capacity to do so. In Canada, recent efforts aimed at facilitating youth engagement in policy have included youth advisory committees at all levels of government and the development of Canada’s first Youth Policy (Government of Canada, 2016, 2018). However, while youth engagement in policy processes has attracted attention across sectors and there is general consensus that including youth in the development of health-related initiatives can yield positive results (Moffat *et al.*, 2013; Jenkins *et al.*, 2016; Nah and Lee, 2016), we currently lack strategies, grounded in evidence, to support this activity.

Given the critical gaps identified, our research team is conducting a study exploring how youth can be meaningfully engaged in the policymaking process to promote mental health and substance use outcomes. This project is unique in focusing on so-called ‘disengaged’ or marginalized youth and on community contexts in which policy participation remains largely inaccessible due to social and structural inequities. Through our early work on this project, we have identified the need for resources to grow youth expertise for impactful policy engagement. This paper presents the findings from our comprehensive environmental scan of youth policy training programmes, which makes an important and timely contribution to the science and practice of youth-driven policy intervention as a mental health promotion strategy.

## MATERIALS

The environmental scanning method originated in the organizational learning field and has since been taken up as a public health needs assessment tool that allows for a rapid and comprehensive mapping of current resources and existing gaps (Rowel *et al.*, 2005; Wong *et al.*, 2010; Wilburn *et al.*, 2016). This approach has been identified as well suited to capturing the contextual factors that contribute to health and social inequities and to informing the development of resources tailored to the needs of communities (Rowel *et al.*, 2005; Carter *et al.*, 2017). As such, environmental scanning is a particularly useful tool for mapping the current landscape of research evidence and community resources that inform capacity-building efforts to support youth to engage in policy processes across a variety of contexts. Furthermore, this approach contributes the flexibility to capture and represent materials that have been produced by governments and the non-profit sector, where many of the resources for building youth capacity for engagement in the policy context are derived.

There is no single method identified for conducting an environmental scan, although Choo’s (Choo, 2001) formative work identified four broad modes of scanning: undirected viewing; conditioned viewing; informal searching, and formal searching. We utilized what Choo referred to as ‘formal searching’, the most systematic and robust scanning approach, which involves active efforts to seek out information and an openness to unanticipated findings. Our process was further informed by Rowel *et al.*’s (Rowel *et al.*, 2005) environmental scan approach, which involves both data searching and engagement of key informants to ensure a comprehensive scan of the context. Our process consisted of two steps: (i) a systematic search of scientific and grey literature and of youth policy-related websites to identify existing resources aimed at building capacity for youth to engage in policymaking processes; and (ii) key informant consultation to refine the search.

### Data sources and search strategy

#### Academic literature search strategy

Two members of the research team (C.A. and M.J.Z.) conducted a parallel search of the academic databases CINAHL, ERIC, MEDLINE, and PsycINFO. This search was performed without date restrictions using the terms: youth, young people, adolescen*, teen*, train*, engage*, educat*, involve*, policy, policy training, policy training course and policy course. The Boolean operators ‘and’ or ‘or’ were applied until all possible combinations were exhausted. When available, MeSH terms and wild cards were applied to allow for greater inclusivity of search results.

#### Grey literature search strategy

Grey literature was also systematically searched by the same two team members using the terms identified above and using Google and Google Scholar. In accordance with the approach developed by the Agency for Healthcare Research and Policy (Porterfield *et al.*, 2010), the first 20 links generated were screened for relevance. When a website relevant to youth policy training was identified, the next 10 links were reviewed until no additional links of relevance were identified.

#### Expert consultation

After the initial searches were complete, preliminary findings were presented to the larger research team, in accordance with Rowel *et al.*’s (Rowel *et al.*, 2005) key informant consultation process. This research team comprises researchers and policymakers with expertise in youth health, youth engagement and youth policy. To facilitate inclusion of a youth perspective, which is often unaccounted for in published materials, our key informant consultation also included interviews with three youth who are employed by a local youth agency and have experience in the research and policy context. This consultation resulted in a further targeted grey literature search that included searching of 10 recommended youth organization websites. Additionally, this process informed a Google search using refined terms: ‘policy training course for youth’; ‘youth policy training course’ and ‘youth engagement in public policy course’.

#### Resource selection

Searching was conducted between January 2018 and April 2018. Searches were limited to studies and websites published in English and focusing on youth up to the age of 30 years, to align with the most inclusive global definitions of ‘youth’. Titles were screened to identify relevant literature. Where a search generated greater than 500 results, title screening was not attempted and new terms were applied to enhance specificity. Inclusion criteria were broad. Academic articles as well as print and online resources developed for government, community or research organizations were reviewed and assessed for applicability (i.e. resources describing strategies to build capacity for youth to contribute to policy). Where programme descriptions lacked information about objectives, curriculum content and outcome evaluation, members of the research team (E.J. and L.M.) contacted the sponsoring organizations to request additional information; however, most were unable to provide details about curriculum development or content (e.g. provided topic headings but no further content details of curricula). Included resources could take multiple formats including seminars, online courses or training ‘toolkits’. Retrieved materials that did not aim to equip youth with the skills to contribute to policy were excluded.

#### Data extraction

Our research team developed a data extraction tool and trained two reviewers (L.M. and C.A.) to extract information on the characteristics of policy training programmes for youth and associated evaluation measures and outcome evidence. The reviewers independently and systematically assessed the programmes identified from both the academic and grey literature. Differences in assessments were discussed and explored iteratively until consensus was reached. Hart’s (Hart, 1992) conceptual model, the ‘Ladder of Youth Participation’, was employed to characterize the degree of inclusion and decision-making power of youth in each programme. The model represents these as eight ladder rungs, where the lowest three rungs involve no or tokenistic participation and the top five rungs involve meaningful participation culminating in projects that are youth-initiated and where youth share decision making with adults.

#### Data synthesis and analysis

A qualitative description approach was used to identify and synthesize the common or important features, including elements of success, across the identified materials (Hyejin *et al.*, 2016).

## FINDINGS

Table 1 presents the number of results generated per database, abstracts/websites assessed, and articles/websites retrieved from the systematic search.

**Table 1:** Results of academic and grey literature search

Academic: databases	Total combined resultsa	Abstracts screened for relevance	Articles retrieved (excluding duplicates)	Grey: titles screened	Resources retrieved (excluding duplicates)
Medline	10 208	17	5		
PsycINFO	20 153	20	1		
CINAHL	7298	7	3		
ERIC	18 987	0	0		
Total	56 646	44	9		
Systematic: google scholar				270	3
Google				260	2
Total				530	5
Targeted: googleTotal				230	20

^a^Includes the total number of results retrieved through each database, including all combinations of search terms.

**Fig. 1:**
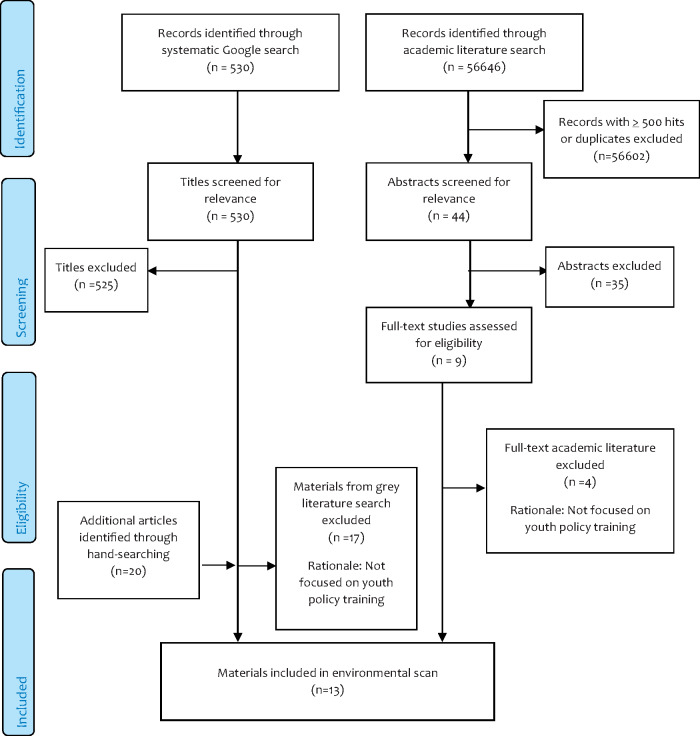
Search process adapted from CONSORT flow diagram (Moher *et al.*, 2009).

Articles and websites identified through the search process were independently screened for relevance by three team members (E.J., L.M. and C.A.). Discrepancies in assessment of relevance were resolved through discussion. Most articles or websites were excluded because they did not include youth or involve policy training specifically. This process resulted in 13 articles and websites identified and retained for data extraction (see Figure 1).

### Characteristics of training programmes

Table 2 summarizes the information extracted from the academic and grey literature for each of the main features of the 13 policy training programmes retained for analysis. Information was extracted about the sponsoring organization and type, location and training/publication date, focus and level of the policy training, target participant demographics including age ranges and other criteria for engagement, and evaluation measures and results. Information was also captured about noted barriers and facilitators and is summarized in the recommendation section below.

**Table 2:** Summary of results from academic and grey literature

Programme name (date)	Country (city)	Participant type, age range and (number)	Training duration, format and programme description	Indicators	Policy focus (level)	Tools (other supports)
**Academic literature results**						
1. Summer Youth Pipeline Project (Rashied-Henry *et al.*, 2012)	USA (New York)	Minority youth aged 15–18 years plus scholastic achievement (51 participants)	4-week summer internship to: increase health disparity knowledge; provide hands-on community-engaged research training; advocate for policy change; encourage health career	Pre–post evaluation surveys including knowledge items	Health inequities (local, regional, national)	(Library orientation, writing templates, ethics course, bus pass)
2. Youth ALIVE! **‘**Teens on Target’ (TNT) (Calhoun, 2014)	USA (California)	High school students in neighbourhoods with high rates of gun violence (900 over 20 years)	Six-session school-based, peer-led workshop Peer education and advocacy violence prevention programme to: train youth as peer educators; lead violence prevention workshops; engage youth to develop public policy solutions to reduce gun violence	Tracked programme activities and policy impacts	Gun violence (local, regional)	TNT violence prevention curriculum, programme and other manuals available http://www.youthalive.org/publications/
3. Neighbour-hoods Working in Partnership (NWP) Project (Cheezum *et al.*, 2013; Israel *et al.*, 2010)	USA (Detroit)	Youth aged 14–22 years and adults (114 youth attended more than 1 session)	Four workshops (3 half-day, 1 city-wide) over 6–8 weeks Community-based participatory research based programme to strengthen policy advocacy skills; involve community members in policy-making, and; promote policies that create safe, supportive neighborhoods	Pre–post evaluation surveys to assess which curricular materials and training activities increased capacity to carry out policy-related work plus individual workshop surveys to assess satisfaction, usefulness	Health inequities(local)	Policy Link developed policy training and provided train-the-trainer workshops https://www.policylink.org/ (Childcare, $10 gift card, food at each session)
4. YMCA Kentucky Teen Institute(King *et al.*, 2015)	USA(Kentucky, multi-site)	Five teams of high school students from diverse communities, mostly White except for one urban-based team, and adult mentors (24 youth)	Yearlong health advocacy intervention to: assess community needs; build prevention capacity; develop strategic plan; implement prevention programmes, policies, practices, and; evaluate outcomes Youth met with adult mentors regularly with support from YMCA and advocacy coach plus 3 events: 5-day summer and 3-day winter events, and 1-day Children’s Advocacy Day	pre–post evaluation surveys: demographic, health and advocacy behavior, health knowledge, Theory of Planned Behaviour construct items; and qualitative analysis of informal/formal interviews, notes and documents	Health(local, regional)	
5. Students Working Against Tobacco (SWAT)(Ross *et al.*, 2015)	USA (Oklahoma)	State middle and high school students	Yearlong school-based campaign, including initial video training on predefined campaign topics and measures of progress (MOPs), designed to: engage youth community action against tobacco cultivate youth-led prevention build youth coalitions	Pre–post evaluation surveys assessing tobacco-related attitudes, self-efficacy, and advocacy behaviours plus youth assessment of campaign materials, structure and implementation process	Tobacco prevention(local)	SWAT training modules 1–11, including tobacco 101, policy change, public speaking, project planning, teamwork, media advocacy http://www.ok.gov/okswat/Be_a_Member/index.html
**1. Grey literature results (A) Seminars/workshops**						
6. European Seminar on Youth Policy-Making (Croatian National Agency, French National Agency & EU-CoE Youth Partnership, 2017–18	Reps from 77 countries	Not limited to youth open to national agencies, youth NGOs and youth movements Age not reported	Multi-day seminars first 3-day residential seminar (concepts and theories) Croatia Reflection phase in own context second residential seminar: 2018 France (reflection and critique) analyse youth policy concepts, strategies, and investigate approaches interrogate principles of youth policy from a variety of perspectives translate policy frameworks to intervention strategies	Indicators not reported	Youth policy (local, regional, national, inter-national)	#12 online course below used in this training
7. Speak Up!(Don Bosco Youth-Net, 2016)	Nine countries (Vienna)	Young people, ages NR (22 participants)	Six-day training course Foster competencies to develop, implement advocacy, especially policies affecting young refugees	Indicators not reported	Social inclusion(local, regional, national, inter-national)	
**1. Grey literature results (B) Courses/toolkits**						
8. Influencing Policy: DIY (YATI—Div’n of The Lung Association)	Canada	Youth aged 12–24 years	Six-hour training seminar to explore how tobacco/nicotine control policy developed, learn how to work with organizations to influence policies to support prevention	Indicators not reported	Tobacco and nicotine (local)	Video resources for youth advocacy
9. Youth Policy in Practice Training Course(Erasmus+: Youth in Action Programme, 2015)	European countries, held in Croatia	Youth workers, youth leaders, project managers, youth policy makers, decision-makers Ages not reported (42 participants)	Four-day training course to explore and encourage cooperation on youth policy issues between local and regional authorities and youth	Indicators not reported	Youth(local, regional)	Not reported
10. Training Course on Youth Participation and Youth Policy (Council of Europe’s Directorate of Youth and the Ministry of Tourism, Culture, Youth and Sports of Albania, 2011)	Albania & European Cultural Convention Countries (Tirana, Albania)	Youth workers, youth leaders, and other practitioners able to work in English ages 18–35 (30 participants)	Six-day training course to foster a participative approach to youth policy development and implementation	Indicators not reported	Youth(local, regional, national, inter-national)	Not reported
11. Youth Advocating for Social Inclusion Policies Training Course (Out of the Box International, Nov 2014)	Bulgaria, Estonia, Hungary, Nether-lands, Denmark, and Portugal	Youth workers/leaders able to work in English Ages 18–35	Four-day training with 4–90 min sessions per day to combine theoretical input with practical learning to empower youth workers with knowledge/skills to participate in youth policy mainstreaming	Indicators not reported	Social inclusion in youth policies(local, regional, national, inter-national)	
12. Essentials of Youth Policy Course (EU-CoE Youth Partnership, October–December 2017)	Open	71% participants aged 19–34, not limited to youth 1805 registered, 558 started course and 208 requested completion certificate	Six module online course requiring intermediate English with 16 facilitated forum discussions to: develop basic competences to engage in youth policy; focus on essential elements; steps of youth policy and; future planning	Pre–post evaluation surveys including knowledge items	Youth (local, regional, national)	Essentials of youth policy YouTube videos http://www.youtube.com/watch?v=_cnTdDoJWAM&list=PLKNmrlD6g-JsNr9hKf9zs0j_Fo9QTVFw5
13. YouthMetre (European Commission funded Forward Looking project co-ordinated by European Association of Geographers, ongoing)	Open	Young people aged 18–30 and those who work with young people	E-toolkit, online Moodle course, or down-loadable files: curriculum, training plan and training materials to empower young people to connect with policy makers and improve youth policies	Indicators not reported	Youth (local, regional, national)	E-toolkit http://youthmetre.eu/etoolkit/ Moodle course https://moodle.eurogeography.eu/login/index.php Downloadable files: http://youthmetre.eu/training/

### General format and timing

All youth policy training programmes identified took place in the USA or the European Union (EU), with the exception of one Canadian program (#8). Apart from two online courses (#12 & 13), training was in-person and most commonly offered in multi-session formats. The duration ranged from a 6-h seminar to weekly sessions spanning the school year. Formats included seminars, multi-day workshops, internships covering several weeks/months and school-based programmes. Programmes that operated over longer periods of time (#4, 5, 6), or were delivered in close collaboration with community partners, offered immediate opportunities for application of acquired skills (#1, 2, 3). In-person programmes utilized interactive learning with peers, and most combined small group discussion/working groups with peer presentations, multimedia and expert guest lectures. Experiential action learning (i.e. role play, reflection, feedback) was featured in one programme (#3). EU programmes employed similar activities but provided time at the outset to build group rapport. An on-line course (#12) provided facilitated forums for participant dialogue and peer-to-peer sharing.

### Geographical differences in programme focus

Interest in building capacity for youth to engage in policy processes has grown over the last decade, with our results illuminating two distinct orientations to policy training for youth that have emerged in North America (NA) and the EU since 2011. The training programmes differ by region in their policy focus, policy level (local/municipal, regional, national, international) and degree of community participation (see Table 2).

NA programmes (#1–5, 8) originated ad hoc as part of community-based health equity projects or as broader health promotion projects. Grassroots projects (#1–3) were organized and delivered locally with a reliance on existing intersectoral partnerships (including academic partners) to involve youth in advancing previously or co-identified community health and safety goals (Israel *et al.*, 2010; Rashied-Henry *et al.*, 2012; Calhoun, 2014). For two (#1, 3), the focus on partnerships stemmed from a community-based participatory research approach, which is predicated on the meaningful involvement of community and academic partners to create community-led change. These programmes were targeted at youth aged 12–18 years—and sometimes other adult community members (#3)—who were either minorities (#1) or lived in areas disproportionately affected by inequities, health among them (#2, 3). Most aimed to impact local community policy although, in one case, larger policy impacts were realized (#2). The Summer Pipeline Project (#1) differs from these and other programmes represented in Table 2 in that the programme had a broader aim to enhance interest among its participants in pursuing a health career and required a level of academic achievement for participation. Health promotion-oriented policy training programmes (#4, 5, 8), two of which were school-based (#4, 5), were organized more broadly by large non-profit organizations as citywide or regional health promotion efforts (King *et al.*, 2015; Ross *et al.*, 2015). Two specifically targeted policies on tobacco (#4, 8) with one featuring pre-made campaigns. These programmes involved community partners later in the process.

In contrast, in EU countries, training was focused on advancing youth policy, featured rights-based approaches and were supported by the EU Youth Strategy (2010–18) with the aim of enhancing more equitable access to education, job and civic engagement opportunities for youth (#6–7, 9–13), including young refugees. Grounded in knowledge of EU youth policy frameworks, these programmes, including two online courses, tended to span policy levels from local through to multi-national. Delivered in various locations in Europe, programmes were organized in partnership with the European Commission’s EU Youth Strategy and its Erasumus+ programme. They included a broad age range of participants (ages 12–35 years) from across the EU nations, including adults experienced in youth policy efforts such as youth workers and/or decision-makers (#6, 9).

### Approaches to curriculum development

Data about how course curricula were developed and taught were limited. More information was provided about who was involved in the development of the training materials, although nearly half did not provide descriptions of the process (#4, 7, 9, 10, 12, 13). Academic partners, programme staff and other experts initially developed the curriculum in three programmes (#1, 3, 5). Youth were involved in the development of only a few programmes (#2, 6, 8), with experts providing support.

Curriculum content, which tended to be available in outline form only, varied widely, with strong geographically-based differences. EU programmes provided grounding in the various levels of youth policy and promoted understanding of how levels interrelate. Most NA programmes focused on education about democratic processes, describing how different levels of government work and how policy change occurs, and alliance and partnership building among stakeholders. In addition, many programmes incorporated elements to build participants’ proficiency in the skills needed to carry out advocacy work, including: how to develop a policy campaign; how to argue effectively in written and oral formats; evaluation; and media strategies. EU programmes included more theory regarding human rights and youth well-being as well as opportunities for peer-to-peer experiential learning.

While information on the level of youth input into programme development and content was limited, combining existing information with details about the format and goals of the programmes permitted an assessment of youth engagement. Four programmes identified in the academic literature (#1, 3, 4, 5) were rated as consistent with level four on Hart’s youth participation scale, where programmes are adult initiated (including the curriculum development), youth volunteer to participate, and youth views are respected. One programme (#2), was adult initiated, but since youth participated in ongoing curriculum development, with adults providing coordination and shared decision making, engagement was assessed as level six, where youth are involved in each step of planning and implementation. This programme also included youth mentors who assisted with teaching and organizing presentations. Most EU programmes were characterized by peer-to-peer mentorship and a clear valuing of youth contribution so were assessed at engagement levels between five (#6, 7) and six (#9). A Canadian training programme (#8) was also assessed at level six because it described youth as being involved in all steps, with training provided ‘in partnership between adult and young adult facilitators’.

### Discrepancy between programme goals and outcome measures

All the NA programmes aimed to develop youth capacity to influence and inform health-related policy. However, programme evaluations focused on short-term, individual participant-level outcomes and quality improvement. Few attempted to track longer-term individual, community or policy-level change. For example, most NA programmes (#1, 3, 4, 5, 8) and one online EU course (#12) conducted formal pre- and post-intervention evaluations that measured individual knowledge gains, attitudes, self-efficacy and advocacy behaviour and intentions (see Table 2, indicators). Of those that undertook evaluations, significant gains were mostly within the domains of knowledge, self-efficacy and confidence in ability to influence policy, as well as increased engagement in advocacy activities. Two programmes (#1, 5) reported lower than expected advocacy activities or intentions. Authors attributed these negative results to project facilitation issues, such as delays in project initiation, a lack of training support, and data collection challenges, rather than a content shortfall.

Grassroots programmes attempted to evaluate broader level community and policy impacts. One programme (#1), where students worked at community-based organizations on local projects, conducted a 4-month follow-up evaluation with community partners to gauge students’ contributions to achieving their goals and found that 70% had used student output to advance their work. Students also reported favourable experiences and ranked this work as their favourite part of the training. Another programme (#2) chose not to evaluate participant outcomes and focused on building trusting relationships with participants. Staff felt that collecting detailed data on minority youth—already conceptualized as ‘high risk’—would jeopardize trust and engagement in the programme. Instead, the programme tracked participation (number of presentations to decision-makers, local and regional partnerships developed) and policy impacts where possible, while acknowledging the difficulty of isolating the variables at play in policy processes. Results were incorporated into the programme and communicated to partners, stakeholders and funders, which increased community support and engagement in the programme. One other, a school-based health promotion training programme (#5) that relied on pre-packaged campaigns, also tracked policy impacts as part of its evaluation processes. Of 30 teams that aimed to create policy change, nine saw policies implemented that were at least partially attributable to the programme.

### Reducing barriers to engagement

NA programme descriptions included a variety of recommendations for recruiting and retaining youth that stemmed from process evaluation results. The health equity programmes emphasized the need to address structural barriers to engagement for marginalized youth, including compensation for their time and expertise, providing food and transit passes, and involving family where appropriate. For all youth, incorporating active, experiential and problem-based learning models informed by a developmental approach (e.g. varying activities frequently, providing flexibility in group and individual task options as well as space for non-productive periods, building in ‘wins’ to sustain interest, and including adult and youth mentorship) were identified as critical to sustaining engagement. Utilizing a variety of delivery methods (e.g. video, text, expert speakers, peer-to-peer learning) was identified as ensuring that diverse learning needs were met. Other engagement enhancing factors included integrating a programme in partnership with a broader community (i.e. alignment of policy target with youth and community goals); incorporating additional training components (e.g. community organizing techniques, group process principles including how youth and adults can effectively work together, leadership development); and inclusion of evaluation to improve the programme and support sustainability.

## DISCUSSION

The importance of addressing youth mental health outcomes through a comprehensive approach, inclusive of mental health promotion, has been identified by experts as a critical path to addressing the significant mental health challenges experienced by youth populations—yet this area remains grossly underdeveloped (Whitehead *et al*., 2014). A key element of a mental health promotion approach involves efforts to intervene at a policy level to fundamentally shift the social and structural determinants that shape youths’ mental health and substance use trajectories and outcomes (Jané-Llopis *et al.*, 2005). Importantly, youth should be at the centre of this process to ensure that policies reflect youths’ lived experiences, and therefore, to maximize relevance and impact (Sinclair, 2006). To date, there has been little guidance on how to best equip youth for meaningful engagement in policy processes, which risks this engagement being tokenistic, or worse, exacerbating inequities by privileging participation by youth with individual social privilege or capacities for engagement. This paper makes a needed contribution to advancing this mental health promotion strategy by presenting key characteristics and facilitators of existing programmes for building youth capacity for policy engagement as well as identifying research priorities to build the evidence-base to guide this practice moving forward.

In this environmental scan, two philosophies can be identified as underpinning capacity building efforts for youth engagement in policy processes—positive youth development and community youth development. There were clear geographically-based preferences to the philosophy taken up, with NA programmes operating from a positive youth development perspective and EU programmes being grounded in a community youth development paradigm. The approaches differ in their conceptualization of youth engagement and decision-making processes (Iwasaki, 2016). Positive youth development perspectives encourage youth engagement as an intervention strategy to facilitate the growth of skills and emotional competency that enhance coping capacity for challenging environments and circumstances and, as such, often focus on those deemed ‘at-risk’ (Zeldin *et al.*, 2001). Community youth development approaches, on the other hand, are grounded in social justice and explicitly intend for youth engagement to effect social inequalities and to improve social outcomes for ‘all’ youth (Iwasaki, 2016). While both approaches recognize the importance of youth engagement in policy processes, positive youth development initiatives have been criticized for their emphasis on individual risk regulation and fear-based constructions of at-risk youth (Riele, 2006) that derive from the dominant cultural representation of youth as vulnerable, troubled and incapable (Checkoway, 2013). Future efforts would benefit from the incorporation of both philosophies in tandem—to inform and capture the benefits of engagement across socioecological domains (Mantoura, 2014).

Incorporating a community youth development lens would require attention to processes and measures to gauge broader social and policy impacts. Few programmes attempted to measure their impact on policy, focusing predominantly on individual youth development outcomes. The complex and processual nature of policy development and change is difficult to track, as noted by authors who attempted to do so, owing to multiple influences and extended time horizons (Israel *et al.*, 2010). However, policy-influence evaluation is a growing field and a number of resources are available to assist with measuring both the conceptual (i.e. changing the ‘thinking’ of key stakeholders) and the instrumental (i.e. changing the ‘actions’ of key stakeholders) impacts of policy engagement (Steinberg *et al.*, 2015) in ways that require few additional resources.

The equity focus advanced by many of the programmes identified through this scan will be critical as efforts to promote meaningful youth engagement in policy continue to grow. Historically, those youth who have had opportunities for policy engagement have often come from contexts of social and structural privilege (Zinck *et al.*, 2013). In an effort to better reflect the needs and expertise of marginalized youth—who are most adversely impacted by policies that fail to account for their everyday lives—strategies that support the meaningful engagement of youth from diverse backgrounds are needed. Despite the equity focus that figured prominently in a number of programmes, a theoretical grounding in critical perspectives or intersectionality was not referenced. An intersectionality approach has been advanced as essential to expanding our understanding of the root causes of inequities and the differential effects of policy on people according to their various and intersecting identities (e.g. culture, gender, socioeconomic status, among others) (Hankivsky and Christoffersen, 2008). According to Lopez and Gadsden (Lopez and Gadsden, 2016), in foregrounding ‘attention on power relations at the individual, institutional and global levels and the convergence of experiences in a given sociohistorical context and situational landscape, [intersectionality] serves as an anchor to advance equity and social justice aims for marginalized communities that have experienced and continue to experience structural inequalities’ (p. 2). Furthermore, this lens informs equity-focused tools, such as the Intersectionality-Based Policy Analysis Framework, to more precisely identify ‘who benefits’ and ‘who is excluded’ from policy goals and priorities that could enhance policy equity (Hankivsky *et al.*, 2014).

In addition to an intersectionality lens, the field of community psychology brings strong theoretical perspectives to inform and advance the equity agenda within youth engaged policy efforts. Community psychology guides a focus on addressing ‘the roots of social problems, empowerment, and the capacity to identify, analyse, and act on issues relevant to youth’ [(Mager and Nowak, 2012), p. 782], with community psychology scholars proclaiming policy engagement to be the ‘best instrument of prevention and promotion’ [(Prilleltensky, 2003), p. 199]. Drawing on community psychology theories, such as the Theory of Sociopolitical Development, which depicts youth policy engagement as a product of social awareness and action moderated by perceived agency and existing opportunity (Watts and Flanagan, 2007), could support the generation of resources that equip youth to appreciate and articulate the social construction of power and inequity. This enhanced capacity for nuanced social analysis would support youths’ policy engagement to effect healthier, mental health promoting contexts and improved mental health and substance use outcomes at a population level.

## LIMITATIONS

There are important limitations to this environmental scan. The publications and resources identified and retained were restricted to those available in English, which may have excluded other programmes and associated outcomes, limiting our understanding of the diversity in programme design and evaluation. In much of the available material, programme descriptions were brief, and thus, the assigned classifications of the level of youth engagement should be interpreted as approximations. The academic literature also presented only a few small studies, limiting the strength of conclusions regarding programme effectiveness. Despite these limitations, the findings provide key learnings to inform the development of evidence-based approaches to equip youth for meaningful policy engagement—a key mental health promotion strategy to more comprehensively address the leading health challenges facing youth today.

## FUNDING

This study was supported by Canadian Institutes for Health Research (Grant number: 153232).
